# Deep learning in magnetic resonance enterography for Crohn’s disease assessment: a systematic review

**DOI:** 10.1007/s00261-024-04326-4

**Published:** 2024-05-01

**Authors:** Ofir Brem, David Elisha, Eli Konen, Michal Amitai, Eyal Klang

**Affiliations:** 1 Faculty of Medicine, Tel Aviv University, Tel Aviv, Israel; 2 Arrow Program for Research Education, Sheba Medical Center, Tel-Hashomer, Israel; 3 Division of Diagnostic Imaging, The Chaim Sheba Medical Center, Affiliated to the Faculty of Medicine, Tel Aviv University, Tel Aviv, Israel; 4 The Division of Data Driven and Digital Medicine (D3M), Icahn School of Medicine at Mount Sinai, New York, NY USA

**Keywords:** MRE, Crohn’s disease, Inflammatory bowel disease, Deep learning, Convoluted neural networks

## Abstract

Crohn’s disease (CD) poses significant morbidity, underscoring the need for effective, non-invasive inflammatory assessment using magnetic resonance enterography (MRE). This literature review evaluates recent publications on the role of deep learning in improving MRE for CD assessment. We searched MEDLINE/PUBMED for studies that reported the use of deep learning algorithms for assessment of CD activity. The study was conducted according to the PRISMA guidelines. The risk of bias was evaluated using the QUADAS‐2 tool. Five eligible studies, encompassing 468 subjects, were identified. Our study suggests that diverse deep learning applications, including image quality enhancement, bowel segmentation for disease burden quantification, and 3D reconstruction for surgical planning are useful and promising for CD assessment. However, most of the studies are preliminary, retrospective studies, and have a high risk of bias in at least one category. Future research is needed to assess how deep learning can impact CD patient diagnostics, particularly when considering the increasing integration of such models into hospital systems.

**Supplementary Information:**

The online version contains supplementary material available at 10.1007/s00261-024-04326-4.

## Introduction

Crohn’s disease (CD) is associated with substantial morbidity [1, 2]. The management of inflammation is crucial in preventing disease complications—emphasizing the importance of effective assessment of inflammation [3].

Colonoscopy stands as the gold standard for CD diagnosis. However, it is invasive, and the evaluation of the small bowel remains inadequate [4]. Magnetic Resonance Enterography (MRE) is a non-invasive technique that is effective for assessment of CD activity [4–8]. MRE’s non-invasive nature also offers potential in evaluating treatment response or identifying therapeutic inefficiency. This can prompt early detection and timely adjustments in therapy to maintain clinical remission [9–11]. Nevertheless, diagnosing Crohn’s disease using MRE is time intensive and demands high expertise.

**Fig. 1:**
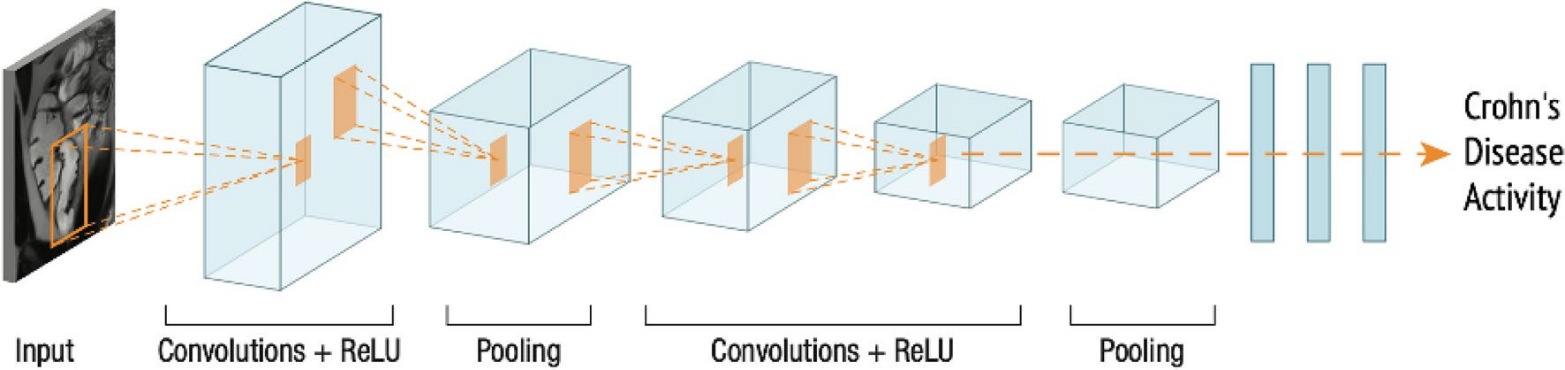
CNN specialize in image processing, utilizing small filters per layer to identify recurring patterns. Their hierarchical structure enables shallow layers to detect low-level patterns and deeper layers to grasp high-level image comprehension

In recent years, Artificial Intelligence (AI), especially convolutional neural networks (CNN), have notably impacted computer vision. CNNs, a type of deep learning (Fig. 1), excel in pattern recognition and are affecting the way in which medical images can be analyzed [12, 13]. This technology offers an innovative approach to diagnosing and monitoring CD activity [14–17].

We reviewed the literature to evaluate articles focused on the use of deep learning to improve MRE analysis in CD.

## Methods

This review adhered to the guidelines outlined in the Preferred Reporting Items for Systematic Reviews and Meta-Analyses (PRISMA). The PRISMA checklist for systematic reviews can be found in Supplementary Materials 1. Studies were selected based on the following systematic approach.

### Search strategy

We conducted an extensive literature search on October 1, 2023, using the PubMed/MEDLINE database to identify studies investigating the use of deep learning for detecting CD in MRI/MRE. Our search terms included “MRI or MRE,” and “Crohn’s disease,” and “deep learning” or related terms, such as “convolutional neural networks,” “machine learning,” and “artificial intelligence.”

Detailed information about the complete search strategies can be found in Supplementary Materials 2. We also conducted a manual search of the references in the studies we included.

Inclusion criteria encompassed studies that (1) assessed the effectiveness of a deep learning model in detecting CD on MRI/MRE, (2) were published in the English language, (3) were peer-reviewed original publications, and (4) included an outcome measure.

Exclusions were applied to articles not related to computer vision, non-deep learning articles, non-original articles, and abstracts.

This study is registered with PROSPERO under the registration number CRD42023484725.

### Study selection

Two authors OB, medical student, and EK, senior abdominal radiologist, autonomously assessed the titles and abstracts to ascertain if the studies satisfied the inclusion criteria. When the title met the inclusion criteria or if any uncertainty arose, a thorough examination of the full-text article was conducted. If a relevant title appeared in the references section of one of the included studies, it was also screened for inclusion. In cases of disagreements, a third reviewer DE, medical student, was consulted for resolution.

### Data extraction

Utilizing a uniform data extraction template in Microsoft Excel, two reviewers (OB and EK) separately gathered information. The data encompassed details, such as publication year, study design and location, patient count, ethical considerations, inclusion and exclusion criteria, study population description, deep learning technique, utilization of an online database, database size, incorporation of an independent test dataset, performance of cross-validation, assessment metrics employed, and the main findings.

### Research questions

The overall aim of this systematic review is to analyze original, peer-reviewed journal publications between 2019 and 2023 on deep learning applications to MRE-based CD assessment. We have defined the following main research questions for our study:

1. What are the different applications of deep learning on MRE in CD?
2. Is there potential for advancement of conventional techniques using deep learning?
3. What are the strengths and limitations of these applications, especially with respect to the field of CD disease burden assessment?
4. What key research gaps are currently being investigated or should be investigated, according to researchers?

### Quality assessment and risk of bias

We evaluated the quality of the studies using the modified Quality Assessment of Diagnostic Accuracy Studies (QUADAS-2) criteria [18]. The task was performed by two researchers: OB and DE. Justifications for the assessment of risk were noted for each criterion in each of the five studies included, and disagreements were resolved by a third researcher (MA, senior abdominal radiologist). No study was excluded based on quality assessment, and it is important to note that four of the five studies had noted “high risk” in at least one category. Details are available in Supplementary materials 2.

## Results

### Study selection and characteristics

**Fig. 2:**
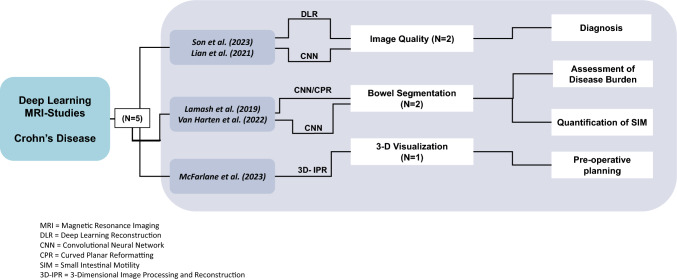
Graphical depiction categorizing deep learning studies according to their clinical application

The initial literature search resulted in 16 articles. Five studies met our inclusion criteria. Studies were published between 2019 and 2023. A total of 468 subjects were analyzed. Table 1 summarizes the characteristics of the included studies. Figure 2 summarizes the clinical application of each study included.

**Table 1:** A summary of articles in the literature review that applied deep learning techniques for magnetic resonance imaging involving Crohn’s disease

Author	Journal	Year	Study design	Database type	Images evaluated by
Son et al.	*Diagnostic Interventional Radiology*	2023	Retrospective	Proprietary	Board-certified abdominal radiologists
Lian et al.	*Results in Physics*	2021	Retrospective	Proprietary	Board-certified abdominal radiologists
Lamash et al.	*Journal of Magnetic Resonance Imaging*	2019	Retrospective	Proprietary	Board-certified abdominal radiologists
Van Harten et al.	*Medical Image Analysis*	2022	Retrospective	Proprietary	Board-certified abdominal radiologists
McFarlane et al.	*Colorectal Disease*	2023	Prospective	Proprietary	N/A

Four of the studies were retrospective, one was prospective. In four of the studies, a board-certified radiologist, served as reference standard. No studies have performed external validation.

### Descriptive summary of results

Studies included in this review utilized deep learning techniques for various tasks.

Son et al. used deep learning-based reconstruction (DLR) and Lian et al. employed CNN to improve image quality. Lamash et al. used CNN for inflammation assessment, while Van Harten et al. measured intestinal motility via centerline segmentation. McFarlane et al. utilized 3D image processing and reconstruction (3D-IPR) with AI to create pre-procedure 3D animations of perianal fistulas (Fig. 2).

Son et al. utilized a DLR technique to enhance the quality of MRE images in Crohn’s patients [18]. A qualitative assessment was carried out by two abdominal radiologists who evaluated three distinct sets of images: (1) original, (2) images processed with a conventional filter, and (3) images processed using the deep learning tool. The mean scores assigned by the radiologists revealed a statistically significant improvement in overall image quality for the DLR image set in comparison to both the original and conventionally filtered images (e.g., Coronal overall image quality: 3.6 (Original), 3.8 (Filtered), and 4.7 (DLR)). Additionally, they conducted a signal-to-noise ratio (SNR) analysis and observed a significant increase in SNR when employing the DLR method.

Lian et al. developed and tested a CNN algorithm with a dataset from 392 patients with epidemic IBD [19]. For the diagnosis of epidemic IBD, they achieved sensitivity 95% and specificity 47%. This demonstrates their CNN algorithm significantly improved image quality.

Lamash et al*.* employed a 1.5-T MRI system and T1-weighted post-contrast VIBE sequence to examine 23 pediatric patients with Crohn’s disease [20]. Their CNN-based segmentation demonstrated Dice Similarity Coefficients of 75% for the lumen, 81% for the wall, and 97% for the background. The median relative contrast enhancement value (*P* = 0.003) demonstrated discriminatory potential between active and non-active disease segments, while various other extracted markers showed differentiation capacity between segments with and without strictures (*P* < 0.05).

Van Harten et al. utilized a deep learning technique for quantification of intestinal motility as a marker of inflammation [21]. They achieved sensitivity 80% and PPV 86% for severe bowel disease with 312 annotated segments between the two groups (185 segments in 14 healthy volunteers vs. 127 segments in the severe bowel disease group consisting of 10 patients).

McFarlane et al. evaluated utility of deep learning-based 3D reconstruction models for 4 perineal CD patients [22]. They found the model provided a more comprehensive visual representation of the disease. No quantitative measures were conducted.

### Quality assessment

As per the QUADAS-2 tool, three papers were identified with a high risk of bias in at least one category. Additionally, none of the five papers discussed in this review have been externally validated. A detailed evaluation of the bias risk is provided in Supplementary Document 2, Table 2.

**Table 2:** A summary of articles in the literature review that applied deep learning techniques for magnetic resonance imaging involving Crohn’s disease

Author	Dataset size	Algorithm	Results
Son et al.	35 patients	Deep learning reconstruction (DLR) to improve image quality	subjective image quality using a 5-point Likert scale (with 5 being the highest quality) the authors found that overall image quality increased with DLR: 3.56 ± 0.54 (original), 3.77 ± 0.56 (filtered), 4.67 ± 0.51 (DLR), *P* < 0.001
Lian et al.	392 patients	CNN algorithm significantly improved image quality	CNN-optimized MRE scans had sensitivity of 95.2% and a specificity of 46.7% in the diagnosis of CD It was found that the optimized CNN algorithm showed higher peak signal-to-noise ratio (PSNR) and structural similarity (SSIM) values and lower high-frequency error norm (HFEN) value in contrast to other algorithms, and the differences were observable
Lamash et al.	23 pediatric Patients with Crohn’s disease (*n* = 17 active disease) (*n* = 6 non-active disease)	CNN used to generate curved planar reformatting (CPR) images of the small bowel	Dice coefficient of 75 ± 18% for the lumen, 81 ± 8% for the wall, and 97 ± 2% for the background when compared with manual boundary delineation Active and non-active disease segments were accurately differentiated (*P* = 0.003) Strictures were accurately identified (*P* < 0.05)
Van Harten et al.	127 MRI segments of 10 patients with pre-operative IBD and 185 MRI segments annotated in 14 healthy volunteers	Deep learning-based automatic untangling of the small intestine and centerline segmentation for quantification of intestinal motility	Dice coefficient of 0.88 ± 0.03 in the set of healthy volunteers and 0.79 ± 0.09 in the set of severe bowel patients
McFarlane et al.	4 patients	CNN to analyze and perform a diagnosis with visual imagery	Prospective study without quantitative results Qualitatively, the authors claim that 3D-IPR improves conceptualization of complex fistulas as it provides a more realistic representation of the anatomy

*DLR* deep learning reconstruction, *CNN* convolutional neural network, *PSNR* peak signal-to-noise ratio, *SSIM* structural similarity, *HFEN* high-frequency error norm

## Discussion

Accurate assessment of CD is pivotal for patient management [23–25]. MRE provides non-invasive insights into both structural and functional aspects without ionizing radiation. Objective endpoints for CD management have been evaluated and established in the form of MRE indices [10, 11, 26]. However, prior research raises concern about MRE analysis being prone to human error, stemming from the time and attention required in radiologists’ interpretations of the entire bowel [27].

The complex nature of CD can benefit from more reliable assessment of disease activity, distribution, and treatment response. This is especially true for clinical trials, where precise quantification of disease is essential. Interestingly, our review demonstrates varied clinical tasks of deep learning applied to CD in MRE. These range from improving image quality, segmenting disease to quantify burden, and 3D reconstruction for surgery planning.

### Image quality

The use of deep learning models presents significant potential advancements in accuracy with these algorithms applied to MRE image analysis for CD [12, 13, 18]. The two included studies in this review evaluated image quality using deep learning applications: Son et al. attempted a DLR technique on 35 patients with known IBD, while Lian et al. employed CNN optimization on 392 patients with suspected IBD. Both studies reported a significantly increased quantitative signal-to-noise ratio. Son et al. demonstrated a qualitative improvement in image quality, including contrast, sharpness, motion artifacts, and blurring in the coronal and axial images; however, DLR image sets appeared more synthetic [18]. Lian et al. demonstrated a lower high-frequency error norm with the optimized CNN algorithm compared to other algorithms and reported a sensitivity of 95.2% and specificity of 46.7% for epidemic IBD, indicating significantly increased image quality with the use of CNN optimization compared to baseline [19].

### Quantification of disease burden

Lamash et al. evaluated the effectiveness of CNN-based segmentation in 23 pediatric patients [20]. Aside from showcasing identification of active from non-active bowel segments, similar to conventional methods, deep learning models were able to accurately, and automatically, identify bowel strictures. Stricture length measurements between two senior radiologists were also reported with greater agreement.

CD, among other gastrointestinal diseases, is associated with deviations in small bowel motility [28–30]. A novel study has emerged for the quantitative assessment of 3D cine-MRI of small intestinal motility in both healthy patients and those with severe IBD [21]. By employing a combination of stochastic tracking and CNN-based orientation classifiers, Van Harten et al. successfully differentiated between motile and non-motile bowel segments. Their findings indicated that such models may surpass the clinically recommended model in assessing ileal CD activity. This highlights the potential for precise, automated, and non-invasive monitoring of intestinal inflammation in CD patients [27].

### 3D image processing and reconstruction

McFarlane et al. developed a CNN-based algorithm to assist in 3D image reconstruction, enhancing surgical planning for four patients with complex perianal fistulas in Crohn’s disease [22]. The reconstruction models provided a comprehensive representation of the perianal disease, aiding in the identification of internal fistula orifices, seton placements, and fistula tracts. While not providing extra information beyond what is acquired through MRI, 3D image reconstruction offers a more realistic depiction of the anatomy. This study demonstrates the potential of CNN-based diagnostic tools to assist surgeons improving surgical outcomes and reducing the number of surgical procedures needed for patients.

The heterogeneous focus of the included studies highlights the broad potential of deep learning in CD assessment via MRE. However, this diversity also complicates direct comparison of outcomes. Despite these challenges, the variety underscores the versatility of deep learning technologies in improving diagnosis, monitoring, and treatment planning for CD. To harness this potential fully, future research must aim for more standardized study designs. Such consistency will facilitate clearer comparisons and enable validation of deep learning applications across clinical settings. This approach will not only streamline research efforts but also accelerate the integration of these technologies into clinical practice, offering new avenues for patient care in CD management.

The heterogeneity observed in the studies under review reflects the multifaceted applications of deep learning across the spectrum of CD management via MRE, highlighting its potential to revolutionize the patient journey in radiology. From improving images through disease burden quantification to treatment planning, deep learning could potentially enhance each step, offering a more nuanced, precise, and patient-centered approach.

Despite the potential on display, research around deep learning is still in its early days, with few studies tackling these varied applications. Analyzing this evolving field of research is crucial. As deep learning and MRE technology advance, they promise to offer deeper insights into CD, enhancing diagnosis, monitoring, and surgical planning. Continuously reviewing these developments is essential for harnessing their full potential to improve patient care.

### Limitations

This systematic review, while providing insights into the emerging role of deep learning in enhancing MRE for CD assessment, has several limitations that warrant discussion:

*Heterogeneity of included studies:* The diversity in the objectives, methodologies, and outcomes of the included studies presents a significant challenge in synthesizing findings. This heterogeneity stems from varied focuses, such as image quality improvement, disease burden quantification, and 3D reconstruction for surgical planning. While this breadth highlights the potential of deep learning across different aspects of CD imaging, it complicates direct comparisons and synthesis of results and prevents performing a meta-analysis. Our rationale for including these diverse studies was to capture this research field’s current directions, acknowledging that deep learning in CD MRE is rapidly evolving with research in its early stages.

*Methodological diversity:* The included studies exhibit a range of study designs, patient populations, and deep learning techniques, which may influence the generalizability and comparability of findings. The predominance of retrospective studies and the absence of external validation in the analyzed research further contribute to potential biases and limit the strength of our conclusions.

*Quality and relevance assessment:* Our systematic review adhered to established guidelines and employed the QUADAS-2 tool for assessing the risk of bias. However, the subjective nature of some assessment criteria and the limited number of studies meeting our inclusion criteria may have impacted the robustness of our evaluation.

Addressing the limitations identified in this review requires an effort toward conducting prospective, multicenter studies with larger sample sizes, and external validation. Standardizing outcome measures and employing consistent deep learning methodologies will facilitate more direct comparisons of findings. Future reviews should also focus on overcoming the challenges of study heterogeneity by exploring specific tasks and specific algorithms of deep learning in CD MRE.

## Conclusion

In this review, we have included a set of preliminary studies evaluating deep learning’s potential to improve conventional assessment of CD MRE. Deep learning models in this field offer promising enhancements to current MRE readings in the tasks of improving image quality, quantification of disease burden, and enhancing surgical planning.

Despite the promise shown by these studies, they are not without limitations. All, but one, of the studies are retrospective and none of them have had external validation. Although this review helps shape our current understanding of deep learning applications in relation to MRE and CD assessment, the findings discussed are limited due to heterogeneity in study design, the small size of the studies included, and the varied clinical tasks explored. As a result of these limitations, the clinical significance of deep learning is still uncertain and in active research.

Future research needs to include direct comparisons between deep learning and conventional radiological assessments. Each of the algorithms discussed must be further evaluated prior to its introduction to the clinical setting. Multicenter prospective studies will be crucial in validating the effectiveness of these systems, thereby establishing their role in the clinical management of CD.

### Supplementary Information

Below is the link to the electronic supplementary material.

Supplementary file1 (DOCX 32 kb)

Supplementary file2 (DOCX 55 kb)

Supplementary file3 (PPTX 52 kb)
