# Supplementary material for: Deep learning in magnetic resonance enterography for Crohn’s disease assessment: a systematic review
Source: Abdom Radiol (NY). 2024 May 1;49(9):3183–9. doi: 10.1007/s00261-024-04326-4 (PMC11335790; doi:10.1007/s00261-024-04326-4)
Supplement: Supplementary file 2 — Supplementary file2 (DOCX 55 kb) [file 261_2024_4326_MOESM2_ESM.docx]

**Supplementary Online Content**

**Title Page.** Outline………………………………………………………………………….…..1

**Supplementary Material 1.** Literature search strategy…………..……………..…..…..…….. 2

**Supplementary Table 1**. Quality Assessment of Diagnostic Accuracy Studies-2......………....3

**References**………………………………………………………………………….……….…..4

This supplementary material has been provided by the authors to give readers additional information about the work.

**Supplementary Material 1: Literature search strategy**

Database: Ovid MEDLINE(R) and Epub Ahead of Print, In-Process & Other Non-Indexed Citations and Daily <1946 to December 28, 2023>

Search Strategy:

--------------------------------------------------------------------------------

1 ("mri" OR "MRE" or "magnetic resonance imaging" or "magnetic resonance enterography”)

2 ("crohn's disease" or "crohn's" or "inflammatory bowel disease")

3 ("deep learning" or "convolutional neural networks" or "machine learning" or "artificial intelligence")

***************************

**Supplementary Table 1: Quality Assessment of Diagnostic Accuracy Studies-2 (QUADAS-2) risk of bias assessment**.

|  |  |  |  |  |
| --- | --- | --- | --- | --- |
| **Author** | **Pt. selection^a^** | **Index test^b^** | **Ref. standard^c^** | **Flow and timing^d^** |
| Son et al. [^1^](https://sciwheel.com/work/citation?ids=15617163&pre=&suf=&sa=0&dbf=0) | 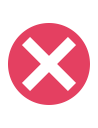 | 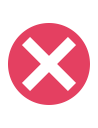 | 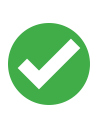 | 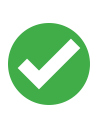 |
| Lian et al. [^2^](https://sciwheel.com/work/citation?ids=15411653&pre=&suf=&sa=0&dbf=0) | 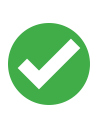 | 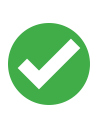 | 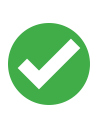 | 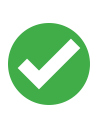 |
| Lamash et al. [^3^](https://sciwheel.com/work/citation?ids=15617153&pre=&suf=&sa=0&dbf=0) | 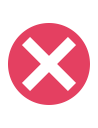 | 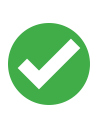 | 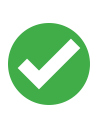 | 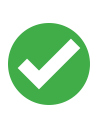 |
| Van Harten et al. [^4^](https://sciwheel.com/work/citation?ids=12983777&pre=&suf=&sa=0&dbf=0) | 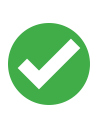 | 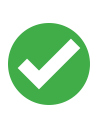 | 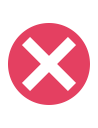 | 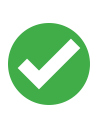 |
| McFarlane et al. [^5^](https://sciwheel.com/work/citation?ids=16191339&pre=&suf=&sa=0&dbf=0) | 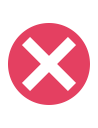 | 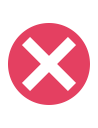 | 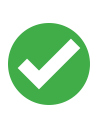 | 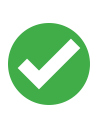 |

Abbreviations: Pt. patient; Ref. reference.
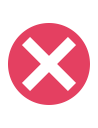
 = high risk of bias;
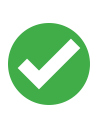
 = low risk of bias.

**References**

[1.    Son JH, Lee Y, Lee H-J, Lee J, Kim H, Lebel MR. LAVA HyperSense and deep-learning reconstruction for near-isotropic (3D) enhanced magnetic resonance enterography in patients with Crohn’s disease: utility in noise reduction and image quality improvement. *Diagn Interv Radiol*. 2023;29(3):437-449. doi:10.4274/dir.2023.232113](https://sciwheel.com/work/bibliography/15617163)

[2.    Lian G, Peng Y, He J, et al. Diagnosis and prognosis of epidemic inflammatory bowel disease under convolutional neural network algorithm and nonlinear equation model. *Results in Physics*. 2021;22:103912. doi:10.1016/j.rinp.2021.103912](https://sciwheel.com/work/bibliography/15411653)

[3.    Lamash Y, Kurugol S, Freiman M, et al. Curved planar reformatting and convolutional neural network-based segmentation of the small bowel for visualization and quantitative assessment of pediatric Crohn’s disease from MRI. *J Magn Reson Imaging*. 2019;49(6):1565-1576. doi:10.1002/jmri.26330](https://sciwheel.com/work/bibliography/15617153)

[4.    van Harten LD, de Jonge CS, Beek KJ, Stoker J, Išgum I. Untangling and segmenting the small intestine in 3D cine-MRI using deep learning. *Med Image Anal*. 2022;78:102386. doi:10.1016/j.media.2022.102386](https://sciwheel.com/work/bibliography/12983777)

[5.    Jeri-McFarlane S, García-Granero Á, Ochogavía-Seguí A, et al. Three-dimensional modelling as a novel interactive tool for preoperative planning for complex perianal fistulas in Crohn’s disease. *Colorectal Dis*. 2023;25(6):1279-1284. doi:10.1111/codi.16539](https://sciwheel.com/work/bibliography/16191339)
