# Supplementary material for: Deep learning in magnetic resonance enterography for Crohn’s disease assessment: a systematic review
Source: Abdom Radiol (NY). 2024 May 1;49(9):3183–9. doi: 10.1007/s00261-024-04326-4 (PMC11335790; doi:10.1007/s00261-024-04326-4)
Supplement: Supplementary file 3 — Supplementary file3 (PPTX 52 kb) [file 261_2024_4326_MOESM3_ESM.pptx]

## Slide 1
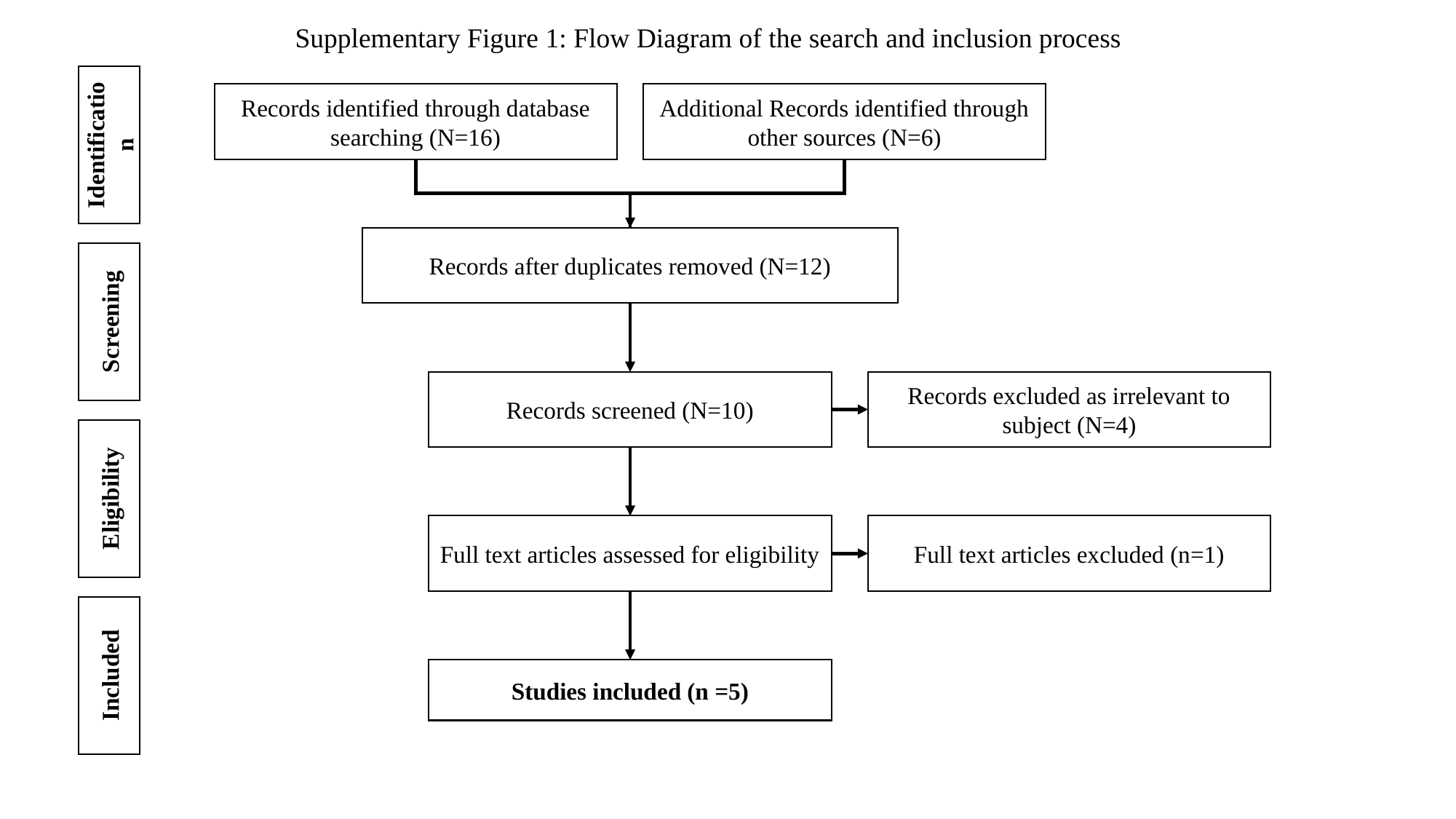

Supplementary Figure 1: Flow Diagram of the search and inclusion process
Identification
Records identified through database searching (N=16)
Additional Records identified through other sources (N=6)
Records after duplicates removed (N=12)
Screening
Records excluded as irrelevant to subject (N=4)
Records screened (N=10)
Eligibility
Full text articles excluded (n=1)
Full text articles assessed for eligibility
Included
Studies included (n =5)

## Slide 2
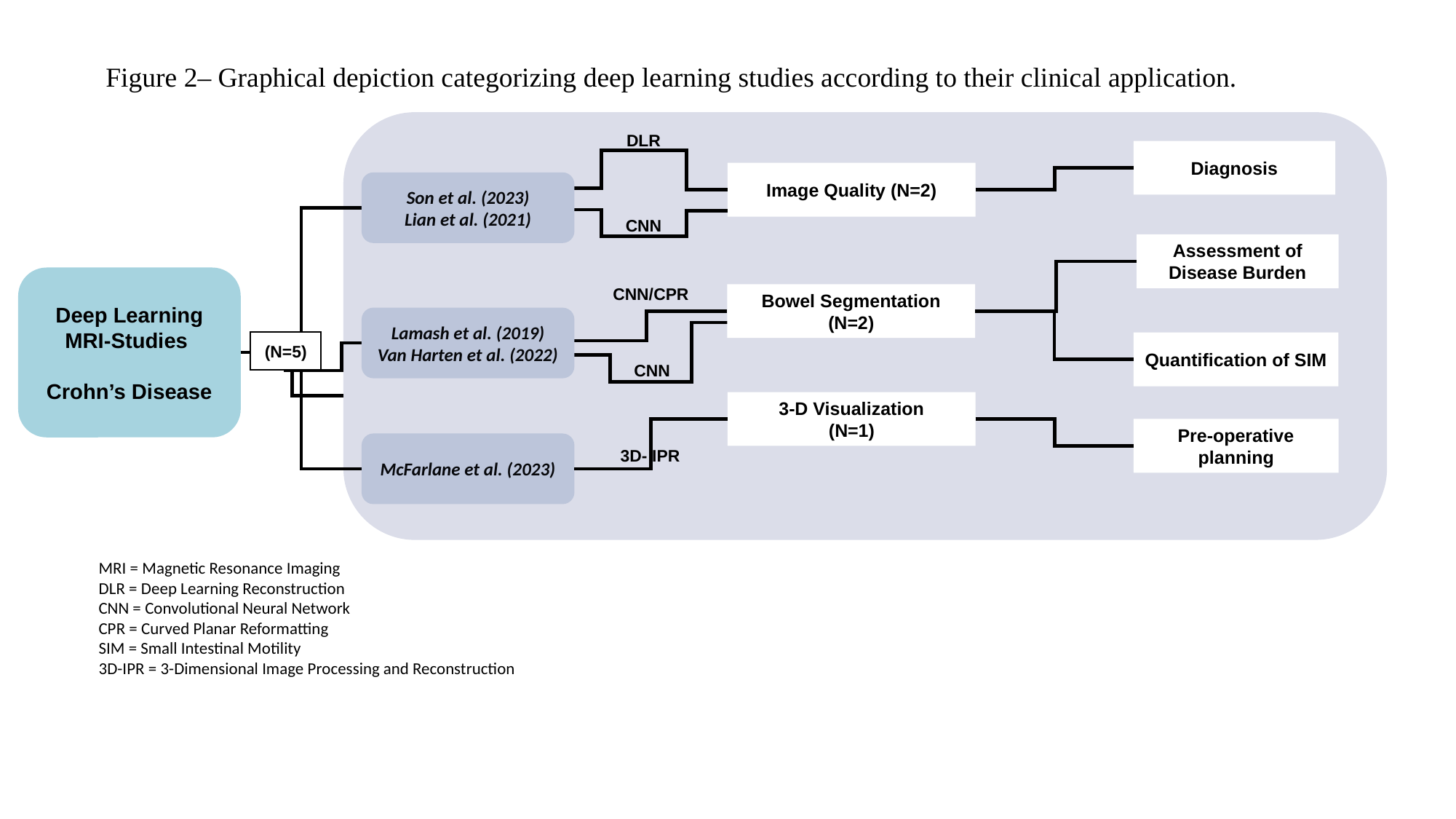

Figure 2– Graphical depiction categorizing deep learning studies according to their clinical application.
DLR
Diagnosis
Image Quality (N=2)
Son et al. (2023)
Lian et al. (2021)
CNN
Assessment of Disease Burden
Deep Learning MRI-Studies
Crohn’s Disease
CNN/CPR
Bowel Segmentation (N=2)
Lamash et al. (2019)
Van Harten et al. (2022)
(N=5)
Quantification of SIM
CNN
3-D Visualization
(N=1)
Pre-operative planning
McFarlane et al. (2023)
3D- IPR
MRI = Magnetic Resonance Imaging
DLR = Deep Learning Reconstruction
CNN = Convolutional Neural Network
CPR = Curved Planar Reformatting
SIM = Small Intestinal Motility
3D-IPR = 3-Dimensional Image Processing and Reconstruction
